# Application of CRISPR/Cas9 in Crop Quality Improvement

**DOI:** 10.3390/ijms22084206

**Published:** 2021-04-19

**Authors:** Qier Liu, Fan Yang, Jingjuan Zhang, Hang Liu, Shanjida Rahman, Shahidul Islam, Wujun Ma, Maoyun She

**Affiliations:** 1 Institute of Agricultural Biotechnology, Jiangsu Academy of Agricultural Sciences, Nanjing 210014, China; qier.liu@murdoch.edu.au; 2 State Agricultural Biotechnology Centre, College of Science, Health, Engineering and Education, Murdoch University, Perth, WA 6150, Australia; Fan.Yang@murdoch.edu.au (F.Y.); J.zhang@murdoch.edu.au (J.Z.); Hang.Liu@murdoch.edu.au (H.L.); shanjida.rahman@murdoch.edu.au (S.R.); s.islam@murdoch.edu.au (S.I.); 3 Triticeae Research Institute, Sichuan Agricultural University, Chengdu 611130, China; 4 Crop Research Institute, Sichuan Academy of Agricultural Sciences, Chengdu 610066, China

**Keywords:** crop, gene-editing, CRISPR/Cas9, quality improvement

## Abstract

The various crop species are major agricultural products and play an indispensable role in sustaining human life. Over a long period, breeders strove to increase crop yield and improve quality through traditional breeding strategies. Today, many breeders have achieved remarkable results using modern molecular technologies. Recently, a new gene-editing system, named the clustered regularly interspaced short palindromic repeats (CRISPR)/Cas9 technology, has also succeeded in improving crop quality. It has become the most popular tool for crop improvement due to its versatility. It has accelerated crop breeding progress by virtue of its precision in specific gene editing. This review summarizes the current application of CRISPR/Cas9 technology in crop quality improvement. It includes the modulation in appearance, palatability, nutritional components and other preferred traits of various crops. In addition, the challenge in its future application is also discussed.

## 1. Introduction

Crop improvement aims to increase crop yield and resistance to biotic and abiotic stress, as well as quality and nutritional value. Crop yield has been significantly increased through advanced agricultural technologies over several decades. Crop quality has been a greater concern of consumers since it is directly associated with human health by providing multiple nutrients such as proteins, fiber, vitamins, minerals, and bioactive compounds [1]. Scientists and breeders have also gradually shifted their focus from increasing production to improving quality. Various strategies have been successfully applied to improve various crop traits, including conventional crossing breeding, chemical- and radiation-mediated mutation breeding, molecular marker-assisted breeding and genetic engineering breeding [2,3,4,5]. However, the conventional mutagenesis-based breeding processes are time-consuming and laborious, especially for polyploid crop breeding [6]. Recently, genome editing (GE) technology which modifies plant genomes in a precise and predictable way, is showing distinct advantages in crop breeding [7].

Genome editing can create predictable and inheritable mutations in specific sites of genome, with the lowest probability of off-target and no integration of exogenous gene sequences. GE-mediated DNA modifications encompass deletions, insertions, single-nucleotide substitution (SNPs), and large fragment substitution. Four site-directed nuclease (SDN) families are involved in a nucleotide excision mechanism: homing endonucleases or mega-nucleases (HEs) [8], Zinc-Finger Nucleases (ZFNs) [9], transcription activator-like effector nucleases (TALENs) [10], and CRISPR-associated protein (Cas) [11]. Most SDNs can accurately target double-strand template DNA to produce a double-strand break (DSB). A plant endogenous repair system automatically fixes the DSBs via two major DNA damage repair mechanisms: nonhomologous end joining (NHEJ) or homologous-directed recombination (HDR). The error-prone NHEJ frequently introduces small indels around the cleavage site, while the HDR precisely repairs the breaks by using the homologous flanking sequence or exogenous repair template, resulting in large insertion or fragment replacement [12]. ZFNs are the first generation of genome-editing nucleases that are generated by combining zinc finger DNA-binding domain with *FokI* endonuclease domain [13]. TALENs consist of a *FokI* cleavage domain and a specific DNA-binding domain from TALE proteins. Comparing with ZFNs, TALENs technology shows a higher target binding specificity and a lower off-target probability [14]. It was widely used as a gene-editing tool in rice [15], wheat [16], maize [15], and tomato [17]. However, both of them require a complex construction process which has constrained their large-scale application in plants. CRISPR was first identified in *E. coli* in 1987 and reported as an immune mechanism to fight against invading viral and plasmid DNA [18]. In recent years, CRISPR/Cas systems have developed to become the most popular GE technology. Compared with other SDNs, the CRISPR/Cas systems are more efficient and straightforward for genome editing because the specificity of editing is dictated by nucleotide complementarity of the guide RNA to a specific sequence without complex protein engineering. Therefore, many researchers have applied CRISPR/Cas tools to gene functional analysis [19]. When introduced into crop improvement field, GE can significantly accelerate the progress of desired traits’ insertion and greatly save labor and other costs. 

The number of cases in crop improvement using GE has increased significantly. Among the various target traits for crop improvement, crop quality is one of the highest objectives. Here, we summarized the recent progress in CRISPR/Cas9-mediated crop quality improvement and provide further discussion on the future application of GE.

## 2. CRISPR/Cas9 Gene-Editing System in Plants

**Figure 1:**
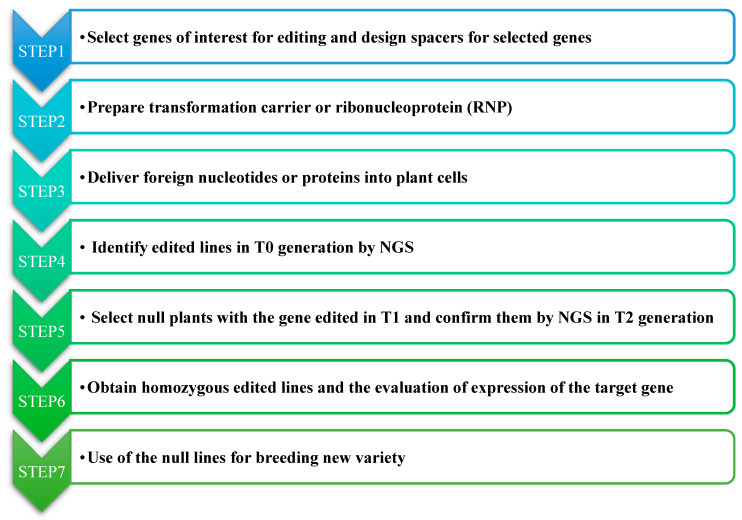
The workflow of CRISPR/Cas9-based gene editing in plants.

According to the classification of the Cas protein, CRISPR/Cas systems have been divided into two classes and five types. The type II CRISPR/SpCas9 system from *Streptococcus pyogenes* has been modified and developed as versatile GE tools for different applications [20]. It consists of two core components: the guide RNA (gRNA or sgRNA) and the Cas9 protein. The gRNA constitutes CRISPR RNA (crRNA) and trans-activating crRNA (tracrRNA). The former contains a ~20 nt fragment (also known as a spacer, complementary to a specific site of target genes), followed by a protospacer adjacent motif (PAM) in the target genes of interest. Under the guidance of gRNA, Cas9 nuclease creates DSBs at ~3 bp upstream of the PAM motif [21]. The cleavage repaired in NHEJ way, usually results in gene knockout or loss of protein function [22]. Alternatively, when an exogenous DNA repair template is provided, HDR can be triggered, resulting in the introduction of the repair template into a target genomic region [23]. In plants, CRISPR/Cas9-based gene-editing consists of multiple steps as shown in Figure 1, including the selection of target sites, designing and synthesis of sgRNA, delivery of transformation carrier or ribonucleoprotein (RNP) in plant cells, transformation, and screening of gene-edited plants. At present, the plant CRISPR/Cas9 and its derived system have shown various genome-editing ability, such as gene knock-in, knockout, knockdown, and expression activation as well. In addition, simultaneous editing on multiple genes have contributed to pathway-level research.

**Figure 2:**
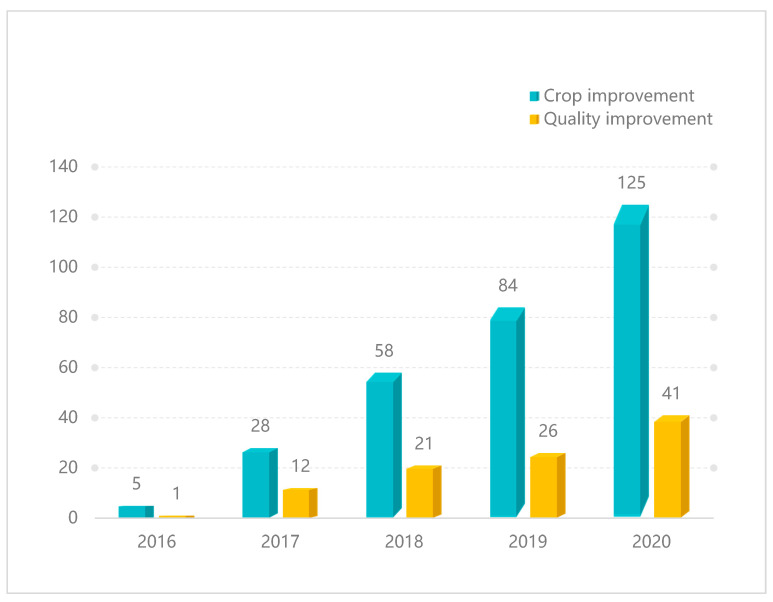
Data on research articles published on CRISPR/Cas9 from 2016 to 2020. ‘CRISPR/Cas9 and crop name’ were used as keywords in the Web of Science search tool (https://webofknowledge.com/) (accessed on 1 April 2021). The literatures aiming at crop improvement were selected and out of them, quality improvement researches were summarized specially, which are shown in blue-green and yellow bar, respectively.

**Figure 3:**
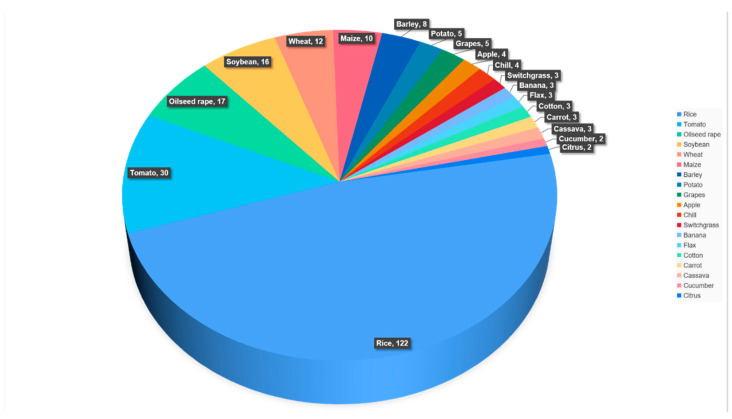
The number of genes modified using CRISPR/Cas system with the aim of crop improvement. Table for the period from 2016 till 2020.

Since the first use of CRISPR/Cas systems for plant gene editing in 2013, many researchers have focused on its application in increasing crop yield, quality, and stress resistance. To date, CRISPR/Cas9-mediated genome editing has been reported in 41 food crop species, 15 industrial crops, 6 oil crops, 8 ornamental crops, 1 fiber crop and feed crop (Table 1) [24]. Furthermore, literature retrieval showed that in the last five years, the number of publications that used CRISPR/Cas9 for crop improvement increased greatly from 5 to 125. Among them, nearly one-third of articles reported improving crop quality by interfering with negative regulatory factors (Figure 2). To demonstrate the extensive application of gene editing in different crop species, we summarized publications on the number of edited genes for each crop species, with the top 3 of those being rice, tomato, and oilseed rape (Figure 3). 

## 3. CRISPR/Cas9-Mediated Molecular Breeding Accelerates Crop Quality 

Crop quality has played a pivotal role in determining the market value of crops. In general, crop quality is determined by external and internal traits. The external quality attributes include physical and aesthetic characteristics, such as size, color, texture, and fragrance. In contrast, the internal quality factors include nutrients (like protein, starch, lipids etc.) and bioactive compounds (such as carotenoids, lycopene, γ-aminobutyric acid, flavonoid and so on). CRISPR/Cas9-mediated crop quality improvement focused on the physical appearance, edible quality, fruit texture and nutritional value (Table 2).

### 3.1. Improving the Crop Physical Appearance 

#### 3.1.1. Modification of Shape and Size

CRISPR/Cas9 technology has been used to optimize the shape and size of the crops according to consumer preferences. Several genes/quantitative trait loci (QTLs) responsible for crop appearance quality have been proposed. The most knowledge on fruit shape and size regulation was revealed in rice and tomato. *GS3* (*GRAIN SIZE 3*), the first QTL identified in regulating grain length, has been successfully knocked out in five japonica rice varieties. The grain length of the T1 lines in all different genetic backgrounds has been increased compared to wild type [25,26]. Grain shape affects not only quality but also grain weight (GW), for example, rice GW has been increased by disruption of multiple grain weight negative regulators, *GW2*, *GW5*, and *GW6* [27]. The role of *TaGW7* has been confirmed to confer an increase in grain width and weight through its knockout in wheat [28]. In horticultural species, researchers can modulate tomato fruit shape and size by modifying the expression of *OVATE*, *CLV* [29], *fas* and *lc* [30], and *ENO* [31]. Among them, *OVATE* and *SUN*, are involved in the asymmetric and symmetric elongation of fruits [32,33]; while *SlWUS* and *SlCLV3* are genes controlling tomato locule number. The gain-of-function mutation of *CLV3* and partially loss-of-function *WUS* are regarded as *fas* and *lc* loci, respectively. Both mutants have positive effects on fruit size [34,35,36]. This has been further confirmed by destructing the cis-regulatory regions of CLV-WUS [30]. 

#### 3.1.2. Color Modification

Plant color is determined by plant pigments composed of carotenoids, anthocyanin, and polyphenols. Especially in plant edible organs, the color of the fruit, leaves, and flower buds affect the consumer’s choice. For instance, Europeans and Americans prefer red-colored tomatoes, while Asian consumers give priority to pink tomatoes [37]. Studies have revealed that the pink phenotype resulted from the absence of flavonoid pigments in the peel. Thus, manipulating the color of fruits can be achieved by disrupting genes involved in the pigment synthesis pathway through CRISPR/Cas9. *MYB12*, as a flavonoid biosynthetic pathway transcription factor, affects the accumulation of flavonoids and governs the pink skin phenotype. Pink-fruited tomatoes have been produced successfully by knocking-out *SlMYB12* [38]. In addition, researchers also created yellow and purple tomatoes by targeting *PSY1* and *ANT1*, respectively. *PSY1* gene encodes phytoene synthase and governs the early steps of carotenogenesis. Mutations in *PSY1* greatly reduced the total lycopene content resulting in yellow flesh tomato fruit [39,40], while the *ANT1*-modified tomatoes enhanced the accumulation of anthocyanins and produced purple plant tissue [41]. In all crop species studied, the anthocyanin biosynthetic structural genes are mainly regulated by *R2R3-MYB*, *bHLH*, and WD-repeat proteins. Knockout of *DcMYB7*, a *R2R3-MYB*, in the solid purple carrot using CRISPR/Cas9 resulted in yellow roots [42]. In ornamental crops, flower color affects the market value, a novel color is always sought after in plant breeders. Several pioneering studies on flower color modification have already been conducted. As a key enzyme participating in flavonoid biosynthesis, flavanone 3-hydroxylase (F3H) is indispensable for the accumulation of anthocyanins. Pale blue flower torenia varieties and pale purplish-pink flowered petunia varieties have been generated by disruption of *F3H* with CRISPR/Cas9 [43,44]. 

### 3.2. Improving Crop Texture Quality 

#### Prolonging Shelf Life

Fruit texture is another key quality in the commercial production of crops. Modifying texture traits for a longer shelf life is an unremitting goal pursued by breeders. The CRISPR/Cas9 technology holds great potential for prolonging the shelf life of tomatoes and bananas. There are several naturally occurring mutant genes with the potential to prolong shelf life, such as *Nr, alc*, *rin*, *nor*, and *Cnr* [45]. However, color absence, undesirable flavor, and low nutritional value accompany these mutations [46]. One study showed that *alc* mutation not only prolonged shelf life but also kept fruit color and fragrance [47]. HDR-mediated gene replacement has been employed to produce tomato *ALC* gene mutations, and the desired *alc* homozygous mutants in T1 generation exhibited excellent storage performance [48]. Another study demonstrated that fruit texture change can be caused by cell wall degrading enzymes [49]. The pectate lyase (PL), known as depolymerase, can disassemble the cell wall during fruit softening [50]. RNA interference of *PL* in tomato exhibited a firmer fruit phenotype [51]. Similarly, CRISPR/Cas9-based knockout mutations of *SlPL* gene exhibited firmer fruit phenotype and longer shelf life without reducing organoleptic and nutritional quality [52,53]. Besides silencing genes that are involved in the degradation of cell walls, downregulate endogenous ethylene production can be another efficient method to delay the fruit softening process [54]. Ethylene is the major factor that affects the post-harvest preservation and shelf life of bananas. *MA-ACO1* is involved in the process of ethylene synthesis and further affects the after-ripening progress [54]. The after-ripening process in *MA-ACO1*-mutant lines has been delayed by about 2 days after ethephon treatment. More interestingly, the content of vitamin C and sugar was increased but no undesired fruit quality happened [55]. 

### 3.3. Improving Palatability

#### 3.3.1. Improving Eating and Cooking Quality 

The eating and cooking quality (ECQ) determines consumer acceptance and also market value. *Waxy* (*Wx*) gene coding for granule-bound starch synthase I (GBSSI) is essential for amylose synthesis. Rice varieties with moderately low amylose content (7–10%) display a soft and sticky texture after cooking, thus being more popular among Asian customers. Several genetic improvement studies have applied CRISPR/Cas9 system to mutate the *Wx* gene in the japonica background rice accessions and successfully produced those with grain amylose content of 5–12% without the penalty on other desirable traits [56,57]. To meet the diverse demands on ECQ, a series of rice mutants with fine-tuned amylose contents have been generated by the precise modification of specific base of *Wx* genes [58]. Meanwhile, waxy maize mutants have been created in twelve elite inbred lines by disruption of the *Wx* gene with CRISPR/Cas9 [59]. Moreover, rice with poor palatability can be attributed to a high grain protein content (GPC) which is negatively related to ECQ. Correspondingly, many elite rice cultivars with satisfactory ECQ normally contain relatively low GPC (usually <7%) [60]. *qPC1* is the first GPC-related QTL that has been identified in rice. An amino acid transporter (*OsAAP6*) in *qPC1* loci functioned as a positive regulator of GPC in rice [61]. Targeted mutagenesis of *OsAAP6* and *OsAAP10* can rapidly reduce GPC and improve the ECQ of rice, providing a new strategy for breeding high ECQ rice cultivars [62].

#### 3.3.2. Improving Flavor

Aroma is another preferred quality trait next to ECQ. Fragrant rice cultivars are popular among rice-eating communities in both Asia and Europe [63]. Research showed that most aromatic rice varieties are especially rich in 2-acetyl-1-pyrroline (2AP) compound [64], which is also important in fresh bread and popcorn and confers popcorn and cracker-like fragrance on food products [65]. Genetic studies have shown the co-segregation of *BADH2* (encoding a betaine aldehyde dehydrogenase) with aroma production [66,67]. It is reported that functional BADH2 participated in the conversion of γ-aminobutyraldehyde (GABald) into GABA, while non-functional mutants of BADH2 convert GABald into 2AP [68]. Therefore, RNAi technology has been used to disrupt *OsBADH2* and further increase the production of 2AP [69]. The first fragrant rice was created by targeting the *OsBADH2* gene using TALENs in 2015 [70]. More recently, researchers have made a breakthrough in creating novel alleles of *OsBADH2* through CRISPR/Cas9, which successfully converted an unscented rice variety, ASD16, into a novel aromatic rice [71].

### 3.4. Biofortification of Nutrient Elements

Consumer preferences are shifting toward healthy and nutrition-enriched food products. Therefore, researchers have been encouraged to create new products to cater for this growing market. Many nutrient elements in vegetables and fruits are effective for anti-inflammatory, anti-cancer, and anti-oxidation. Breeding programs have been implemented on biofortification of diverse nutrients including carotenoid, γ-aminobutyric acid (GABA), iron and zinc contents in various crops. It has been tried to satisfy the “hidden hunger” with quality nutrients through gene-editing for biofortification.

#### 3.4.1. Increasing Carotenoid Content

Carotenoids have been involved in antioxidant processes and eye-related disease prevention. However, humans cannot synthesize carotenoids and must ingest them from their diet. In addition, lycopene and phytoene help to reduce the risk of cancer and cardiovascular disease. Previously, researchers simultaneously introduced *CrtI* and *PSY* genes and synthesized β-carotene in rice through classical genetic engineering. However, such genetically modified (GM) golden rice induced public panic under a strict GM regulatory regime. Many anti-GMO activists insist that this project seems too idealistic, as golden rice may not provide enough β-carotene to eradicate vitamin A deficiency; in addition, the potential risks of planting and consuming golden rice include allergies or antibiotic resistance. There is also a possibility that GMO crops could negatively impact the environment and biodiversity [72]. CRISPR/Cas9-mediated genome editing has been applied in carotenoid biofortification in rice, tomato, and banana. Those produced by this strategy are promising to escape from a GM regulatory regime due to no exogenous gene integration in host genomes. Generally, two kinds of strategies were used for carotenoid biofortification. First, overexpression of phytoene synthase genes through CRISPR/Cas9-mediated knock-in imposes carbon flux into the carotenoid biosynthetic pathway. By this, a carotenogenesis cassette containing *CrtI* and *PSY* genes has been integrated into the target site in rice, resulting in marker-free gene-edited mutants containing 7.9 μg/g β-carotene in dry weight [73]. Another strategy is to block the conversion of their precursors or through silencing corresponding genes, such as (*LCYe*, *BCH*, *ZEP*, and *CCD4*). For example, a golden fruit banana mutant with β-carotene-enriched up to six-fold was created via disruption of the *LCYe* gene [74]. Similarly, the lycopene-enriched tomato was created by disruption of five carotenoid metabolic-related genes (*SGR1*, *LCYe*, *BLC*, *LCY-B1*, and *LCY-B2*) with a five-fold increase in lycopene content [75]. 

#### 3.4.2. Increasing γ-Aminobutyric Acid Content

GABA is a non-protein amino acid inhibitory neurotransmitter, functioning in anti-anxiety and blood pressure control system [76]. Therefore, developing new GABA-rich foods has become the focus of the food industry. The glutamate decarboxylase (GAD) is a key enzyme catalyzing the decarboxylation of glutamate to GABA. GAD has a C-terminal autoinhibitory domain, which negatively regulates GAD activity. In order to increase the content of GABA, the C-terminal has been deleted completely using CRISPR/Cas9. The accumulation of GABA in mutant tomatoes increased seven-fold [77]. Furthermore, researchers have also created GABA-rich rice by truncating the C-terminal of the *OsGAD3* through CRISPR/Cas9 system and the GABA content increased seven-fold [78]. Undoubtedly, GABA-rich crops have a beneficial effect on human health. However, blindly pursuing high content of GABA could not only provoke a reduction in glutamate but also lead to a defective phenotype in fruit [79]. Li et al. (2018) used a multiplex CRISPR/Cas9 method to delete *SlGABA-Ts* and *SlSSADH*, which resulted in GABA levels increasing by about 20-fold but with accompanying high penalties in tomato fruit size and yield [80]. 

#### 3.4.3. Biofortification of Micronutrients

Around two billion people currently suffer from the deficiency of micronutrients, like selenium, zinc, iron and iodine. Biofortification of crop plants with micronutrients would be a sustainable approach for those people who endure an unbalanced diet. In rice, the potential example to use CRISPR/Cas9 method is to knockdown *Vacuolar Iron Transporter* (*VIT*) genes, such as *OsVIT2,* to achieve the increase of Fe content in grain. In a recent study, mutation of *OsVIT2* resulted in increased Fe distribution to embryo and endosperm of the grains, and eventually increased Fe content in the polished grain without negative effect on yield [81]. In addition, the gain-of-function *arsenite tolerant 1* (*astol1*) mutant of rice significantly increased the grain content of selenium (Se), an essential micronutrient with antioxidant effects for humans. The development of micronutrient-enriched rice and wheat grains can also benefit from gene-editing approach by regulating the expression of genes involved in ion homeostasis [82]. 

#### 3.4.4. Improving Fatty Acid Composition

Monounsaturated fatty acids (MUFA), like oleic acid (18:1), are found in abundance in olive oil. Diets rich in oleic acid have favorable cardiovascular benefits. Their counterparts, saturated fatty acids and trans-fatty acids are often listed as “unhealthy” fats and linked with cardiovascular disease [83,84]. Soybean oil as the most widely produced and consumed edible oil, and contains only 20% oleic acid, much less than that in olive oil (65–85%) [85]. Several fatty acid desaturase genes, such as *FAD2* and *FAD3*, were targeted and mutated for regulating the fatty acid composition in soybean. In 2019, researchers had already increased the oleic acid levels from 20% to 80% by editing two homeologous genes of *GmFAD2*, while the linoleic acid level dropped from 50% to 4.7% [86]. Similar breeding strategies have been conducted in rapeseed and camelina with the oleic acid content increasing by 7% and 34%, respectively [87,88]. Recently, the first gene-edited high oleic soybean line has been commercialized for sale in the United States market, with 80% oleic acid and up to 20% less saturated fatty acid [89].

#### 3.4.5. Eliminating Anti-Nutrients

Several substances have negative effects on the nutritional quality of crops, such as phytic acid, gluten protein, and cadmium (Cd). Genome editing can also be used to decrease undesired substances. Humans are unable to metabolize phytic acid due to the lack of corresponding degrading enzymes. When substantial phytic acid is ingested by humans, the absorption of minerals and protein will be reduced since phytic acid can bind with them to form complexes [90]. In order to reduce the phytic acid content in rapeseeds, an *ITPK* gene encoding an enzyme that catalyzes the penultimate step of phytate synthesis has been knocked out by CRISPR/Cas9 [91]. The *ITPK* mutants exhibited a 35% reduction in phytic acid without change in plant performance [92]. In addition, the gluten proteins in wheat can trigger coeliac disease in gluten intolerance individuals [93]. Conventional breeding methods can hardly reduce gluten content, because of the more than 100 loci coded for gluten protein in the wheat genome. Using CRISPR/Cas9 to target a conserved region of the α-gliadin genes, the low-gluten, transgene-free wheat lines have been created [94]. Moreover, CRISPR/Cas9 technology has facilitated the breeding of heavy metal pollution-safe rice cultivars. Cd has been classified as a human carcinogen, the long-term intake of Cd-contaminated rice can cause chronic disease, such as renal failure and cancer [95]. Therefore, creating low-heavy-metal rice in Cd-contaminated areas is a challenge for scientists [96]. By mutating *OsNramp5*, which mediates the root uptake of Cd, researchers developed new Indica rice lines with low Cd accumulation in grain. Moreover, the agronomic traits and the grain yield of *osnramp5* mutants were unaffected when grown in high Cd conditions [97].

**Table 2:** List of research on crop quality improvement by using CRISPR/Cas gene-editing technology.

Application	Crop	Editing Effector	Target Gene	Associated Trait	References
**Physical and appearance quality**	Rice	Cas9	*GS3*, *Gn1a*	Grain length	[25]
		Cas9	*GW2*, *GW5*, *TGW6*	Grain length and width	[27]
		ABE	*GL2/OsGRF4*, *OsGRF3*	Grain size	[98]
		Cas9	*GS9*	Slender grain shape	[99]
		Cas9	*GW5*	Grain width	[100]
		Cas9	*OsGS3*, *OsGW2* and *OsGn1a*	Grain length and width	[101]
	Tomato	Cas9	*ANT1*	Fruit color (purple)	[17,41]
		Cas9	*SlMYB12*	Fruit color (pink)	[102]
		Cas9	*CRTISO*	Fruit color (tangerine)	[103]
		Cas9	*Psy1*, *CrtR-b2*	Fruit color (yellow)	[104]
		Cas9	*OVATE*, *Fas*, *Fw2.2*	Fruit size, oval fruit shape	[29]
		Cas9	*fas*, *lc*	Fruit size	[30]
		Cas9	*ENO*	Fruit size	[31]
		Cas9	*CLV3*	Fruit size	[29]
	Wheat	Cas9	*TaGW7*	Grain shape	[28]
		Cas9	*TaGW2*	Grain size	[105]
	Maize	Cas9	*Psy1*	Seed color	[106]
	Carrot	Cas9	*DcMYB7*	Root color	[42]
	Groundcherry	Cas9	*ClV1*	Fruit size	[107]
	Kale	Cas9	*CRTISO*	Yellow leaves and stems	[108]
	Ipomoea nil	Cas9	*CCD*	Flower color	[109]
	Fournieri	Cas9	*F3H*	Flower color	[43]
	Petunia	Cas9	*F3H*	Flower color	[44]
	Petunia	Cas9	*Ph ACO*	Flower longevity	[110]
**Texture, palatability quality**	Tomato	Cas9	*ALC*	Long shelf life	[48]
		Cas9	*PL*, *PG2a*, *TBG4*	Long shelf life	[53]
	Banana	Cas9	*MaACO1*	Long shelf life	[55]
	Rice	CBE	*OsGBSSI*	Low amylose content	[58]
		Cas9	*OsGBSSI*	Low amylose content	[111]
		Cas9	*OsAAP6*, *OsAAP10*	Reduce GPC	[62]
		Cas9	*OsBADH2*	Fragrant rice	[71]
	Maize	Cas9	*SH2*, *GBSS*	Supersweet and waxy corn	[112]
		Cas9	*Wx1*	Waxy corn	[59]
	Barley	Cas9	*HvGBSSIa*	Low amylose content	[113]
	Potato	CBE	*StGBSS*	Low amylose content	[114]
	Sweet potato	Cas9	*IbGBSSI*	Low amylose content	[115]
	Cassava	Cas9	*PTST1*, *GBSS*	Low amylose content	[116]
**Nutritional quality**	Rice	Cas9	*OsBEI and OsBEIIb*	High amylose content	[117]
		Cas9	*CrtI*, *PSY*	High β-carotene content	[73]
		Cas9	*OsGAD3*	High GABA content	[78]
		Cas9	*OsNramp5*	Low Cd accumulations	[97]
		Cas9	*OsFAD2-1*	High oleic acid proportion	[118]
		Cas9	*OsPLDα1*	Low phytic acid content	[119]
	Tomato	Cas9	*SlGAD2*, *SlGAD3*	High GABA content	[77]
		Cas9	*slyPDS*	Increased lycopene content	[75]
	Rapeseed	Cas9	*BnFAD2*	High oleic acid proportion	[88]
		Cas9	*BnITPK*	Low phytic acid content	[92]
		Cas9	*BnTT8*	High oil production and GPC	[120]
	Camelina	Cas9	*CsFAD2*	High oleic acid proportion	[87]
	Wheat	Cas9	*α-gliadin genes*	Low gluten content	[94]
	Potato	Cas9	*StSBE1*, *StSBE2*	High amylose content	[121]
	Sweet potato	Cas9	*IbGBSSI*, *IbSBEII*	High amylose content	[115]
	Grape	Cas9	*ldnDH*	Low tartaric acid	[122]

Note: List of research on crop quality improvement.

## 4. Challenges and Future Perspectives

At present, the development of gene editing in crops is much more rapid than that in other fields. As shown in Table 1, many quality-related traits have been successfully modified and improved in various crops by the CRISPR/Cas9 technology. Even some gene-edited crops have been commercialized, such as TALEN-*fad2* soybean, TALEN-*ppo* potato, and CRISPR-*wx1* maize, however, we are still at the beginning of this gene-editing revolution. 

To accelerate gene-edited crop commercialization, priority should be given to addressing the policy and technical limitations. First, the policies and regulations of gene-edited crops are controversial and ambiguous worldwide, as different countries have different regulatory frameworks. For most countries, the development and commercialization of new gene-edited crops is mainly subject to the genetically modified organisms’ (GMO) regulatory frameworks. The USA as well as some South American countries, such as Argentina, Brazil, Chile, and Colombia, have employed similar product-based regulations that gene-edited products would be exempt from GMO supervision if the final products have no exogenous DNA [123,124]; whereas the European Union (EU) and New Zealand have strict process-based regulations for genome-edited crops resulting in expensive and time-consuming GM safety tests. China also relies on a process-based GMO regulatory system, as any gene-edited crops are subject to strict scrutiny and no gene-edited crop has been commercialized yet. Under such strict regulation, the advantages of genome editing have been eliminated. Therefore, it is critical to establish a globally unified and specialized regulatory system for genome-edited crops. Recently, 13 World Trade Organization members issued a statement supporting the use of gene editing in agricultural innovation; this was the first step towards establishing a worldwide regulatory framework [125].

In addition, the delivery of CRISPR/Cas9 cargoes would be the thorniest problem for the utilization of plant gene-editing technology. Especially in monocots, biolistic bombardment and *Agrobacterium*-mediated transformation, efficiency is greatly affected by the recipient genotype. For example, some elite rice cultivars are usually difficult to be transformed due to lack of the characteristics suitable for culture and regeneration [126]. Moreover, the integration of T-DNA is unavoidable and the subsequent plant regeneration procedures are often technically demanding and laborious. Therefore, developing no tissue culture-required delivery methods is desirable, with its application further broadened to various plant species. Nanomaterials, such as carbon nanotube (CNT) and nanoparticles (NPs), enable gene or plasmid DNA to diffuse into walled plant cells without any external force or aid, which displays a promising application in CRISPR/Cas9 system [127]. In 2017, “pollen magnetofection”, a novel method using magnetic NPs as DNA transporters, was used to deliver exogenous genes into pollen grains of several model crops. After pollinating with magnetofected pollen, about 1% of transgenic plants were generated [128]. However, some scientists doubted the reproducibility of pollen magnetofection [129]. If CRISPR/Cas9 cargoes can be transported to reproductive cells and stably expressed through pollen magnetofection method, it will be a shortcut to create heritable gene modification in transgenic seeds without tissue culturing [130]. In addition, due to the non-integrating and non-pathogenic performances of the nano delivery tools, the nanomaterial-mediated gene-edited crops may be excluded from GMO [131].

Another concern is the specificity of plant CRISPR/Cas9 systems for targeted gene editing. Some studies have indicated that CRISPR/Cas systems have off-target activity of great potential and sgRNA/Cas9 complexes could cause mismatched DNA sequences in mammals [132,133]. Nevertheless, the results of whole-genome sequencing revealed that the frequency of off-target mutation induced by CRISPR/Cas9 in plants is quite low [134]. Occasional off-targeting can be an issue in gene functional studies since it may affect the phenotype of interest and lead to the inaccurate interpretation of results. However, when using CRISPR tools in crop breeding, the effect of off-target can be ignored [135]. Since off-target mutations with negative effects on phenotype will be discarded during the breeding process, beneficial off-target mutations can be kept in descendants. Therefore, screening beneficial mutations is more important than identifying off-target mutations in the breeding of gene-edited crops. Several strategies have been proposed to minimize off-targeting. Firstly, the majority of off-targeting can be eliminated by designing highly specific sgRNAs with the lowest number of predicted off-targets [136]. Secondly, the specificity of CRISPR systems can be enhanced by using high-fidelity Cas9 enzymes, such as eSpCas9 [137] and SpCas-HF [138]. Finally, the ribonucleoprotein (RNP) delivery method can be used to reduce the exposure duration of the genomic DNA to the CRISPR reagents, thus lowering off-targeting rates [139].

## 5. Conclusions

The advent of the CRISPR/Cas9-based gene-editing tool provides researchers with the ability to modulate crop-specific traits in a more precise and effective way. The CRISPR/Cas9 system has become the most used and versatile technology in crop breeding and functional genomics. With the incomparable capability to modulate genes, it helped create numerous crop varieties with desired agronomic performances. However, most gene-editing work aiming at crop improvement is still at a stage of elucidating the genomic function and regulatory mechanisms. The commercialization of gene-edited crops still has far to go. In addition, gene-editing tools have not met all the requirements for plant genome editing. Further improvement will be crucial for the utilization of CRISPR/Cas in plants as some quality-related traits are controlled by many QTLs and regulating individual genes may not cause significant phenotypic change. It would be feasible to develop an efficient CRISPR/Cas-mediated chromosome rearrangement method. In addition, the delivery of CRISPR cargoes is still a major obstacle. Thus, developing novel carrier materials would be desirable. Besides those, public concerns and government strict regulatory policy of gene-editing technology are another obstacle to innovations in plant breeding. Despite the remaining challenges that need to be resolved, it is believed that gene-editing technology will be more widely used in future and will inevitably play an important role in crop quality improvement.

## Figures and Tables

**Table 1:** Summary of gene-edited crop species using CRISPR/Cas9 system.

Crops in Six Categories	Species
Feed Crops	Alfalfa
Fiber Crops	Cotton
Food Crops	Apple, Banana, Barley, Basil, Blueberry, Cabbage, Carrot, Cassava, Chickpea, Chill, Citrus, Coconut, Cowpea, Cucumber, Date Palm, Grapefruit, Grapes, Kale, Kiwifruit, Lactuca sativa, Lemon, Lettuce, Lychee, Maize, Melon, Oats, Orange, Papaya, Pear, Pepper, Potato, Pumpkin, Rice, Saffron, Strawberry, Sugar beet, Sweet potato, Tomato, Watermelon, Wheat, Yam
Crops for Industrial Use	Cichorium intybus, Coffee, Dandelion, Hevea brasiliesis, Jatropha curcas, Millet, Papaver, Parasponia, Salvia miltiorrhiza, Sorghum, Sugarcane, Switchgrass, Tragopogon, Tripterygium wilfordii
Oil Crops	Canola, Flax, Oil palm, Oilseed rape, Soybean, Sunflower
Ornamental Crops	Lily, Lotus, Petunia, Poplar, Rose, Sedum, Snapdragon, Torenia fournieri

## Data Availability

Not appliable.
